# Impact of the Resistance Responses to Stress Conditions Encountered in Food and Food Processing Environments on the Virulence and Growth Fitness of Non-Typhoidal *Salmonellae*

**DOI:** 10.3390/foods10030617

**Published:** 2021-03-14

**Authors:** Silvia Guillén, Laura Nadal, Ignacio Álvarez, Pilar Mañas, Guillermo Cebrián

**Affiliations:** Departamento de Producción Animal y Ciencia de los Alimentos, Facultad de Veterinaria, Instituto Agroalimentario de Aragón-IA2-(Universidad de Zaragoza-CITA), 50013 Zaragoza, Spain; silviaguillen@posta.unizar.es (S.G.); lnadal@unizar.es (L.N.); ialvalan@unizar.es (I.Á.); manas@unizar.es (P.M.)

**Keywords:** *Salmonella*, foodborne pathogen, food preservation, stress resistance responses

## Abstract

The success of *Salmonella* as a foodborne pathogen can probably be attributed to two major features: its remarkable genetic diversity and its extraordinary ability to adapt. *Salmonella* cells can survive in harsh environments, successfully compete for nutrients, and cause disease once inside the host. Furthermore, they are capable of rapidly reprogramming their metabolism, evolving in a short time from a stress-resistance mode to a growth or virulent mode, or even to express stress resistance and virulence factors at the same time if needed, thanks to a complex and fine-tuned regulatory network. It is nevertheless generally acknowledged that the development of stress resistance usually has a fitness cost for bacterial cells and that induction of stress resistance responses to certain agents can trigger changes in *Salmonella* virulence. In this review, we summarize and discuss current knowledge concerning the effects that the development of resistance responses to stress conditions encountered in food and food processing environments (including acid, osmotic and oxidative stress, starvation, modified atmospheres, detergents and disinfectants, chilling, heat, and non-thermal technologies) exerts on different aspects of the physiology of non-typhoidal *Salmonellae*, with special emphasis on virulence and growth fitness.

## 1. Introduction

Foodborne pathogens have had to develop resistance mechanisms that enable them to withstand stressful environmental and processing conditions they face along the food chain and just before reaching the gut, such as starvation and acidic stomach conditions [1,2]; they have also had to modify and fine-tune their virulence mechanisms in order to evade their host’s defense systems [3,4] in a co-evolutionary process with the latter. In this sense, although the differences between those two evolutionary adaptation processes (adaptation to environmental stresses and to hosts) are obvious, they have several characteristics in common. Both processes enable bacteria to survive under adverse conditions, and both involve changes in common molecules and structures such as the cell wall, the bacterial membranes, and their proteins (porins, efflux pumps, etc.). In fact, it has been proved that microbial pathogenesis relies to a great extent on the ability of bacteria to cope with stresses beyond those imposed by the stomach’s low pH, such as resistance to oxidative stress [5]. This indicates that there is an intimate relationship between virulence and stress resistance, on the one hand, and highlights the complexity and fine tuning of bacterial gene expression regulation systems on the other. It is also well known that the development of stress resistance can impose a fitness cost to bacteria (as demonstrated for *Escherichia coli rpoS* expression), or as a consequence of the acquisition of resistance to certain antimicrobials [6,7]. This aspect is of utmost relevance for food safety, since the bacterial ability to grow and compete for nutrients in foods would determine, along with stress resistance, the number of viable cells reaching the gut, and thus, capable of causing illness.

*Salmonella* is a very good example of successful evolution and adaptation to different niches and hosts. Non-typhoidal *Salmonella* serovars are the second most frequent zoonotic agent in the European Union and the United States [8,9], and are now regarded as a re-emerging pathogen [10]. The *Salmonella* genus includes two species, *S. bongori* and *S. enterica*, whereby the latter is divided into six subspecies. Among those subspecies, *S. enterica* subsp. enterica is a foodborne bacterial pathogen with at least 2600 serotypes [11]. Because of this remarkable genomic diversity, *Salmonella* is found in complex environmental and ecological niches, and survives in harsh environments for long periods [12,13]. This makes it complicated to reduce its overall incidence because *Salmonella* has many sources, which vary according to serotype [14]. Such a degree of genomic, niche, and host diversity makes *Salmonella* a very good model to study the relationships between microbial stress resistance, virulence, and growth fitness.

**Figure 1:**
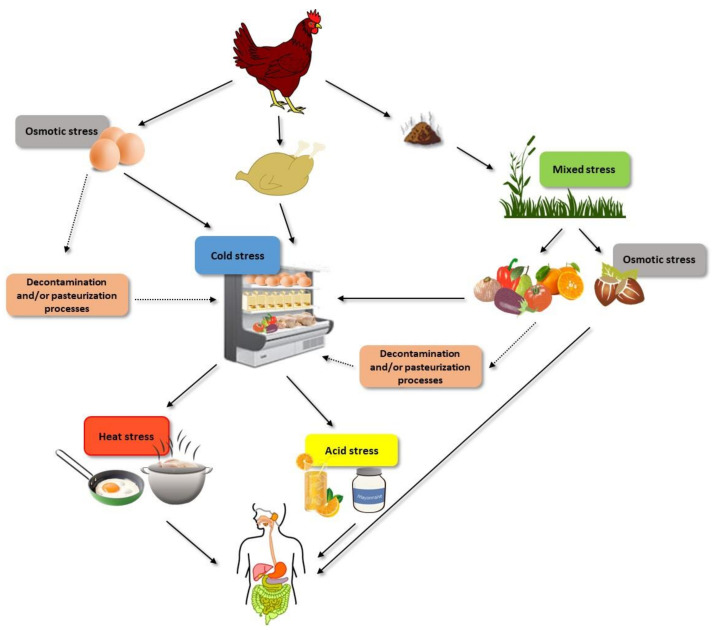
Examples of the different stresses that non-Thyphoidal *Salmonella* cells can face before being ingested with food.

The objective of this review is therefore to provide a summary of current knowledge regarding the effect that microbial resistance responses to different agents faced by non-Thyphoidal *Salmonella* within the food chain (Figure 1)—including acid, osmotic and oxidative stress, starvation, modified atmospheres, detergents and disinfectants, chilling, heat, and non-thermal technologies—might have on other aspects of microbial physiology, with special emphasis on virulence and growth fitness. The impact of resistance to antibiotics and to other chemical agents such as essential oils and/or natural antimicrobials on *Salmonella* virulence and growth fitness will not be discussed in this review. Information on those particular agents can be found elsewhere [4,15].

## 2. Bacterial Stress Resistance, Virulence, and Growth Fitness

Environmental stress can be defined as an external factor that exerts an adverse effect on the physiological welfare of bacterial cells, leading to reduction in growth rate, or, in more extreme circumstances, to inhibition and/or death at individual cell or population levels [16]. In the food processing environment, these stressing factors include agents of a very different nature, including chemical and physical agents such as low pH, low water activity, heat, pulsed electric fields, and ionizing radiation.

**Figure 2:**
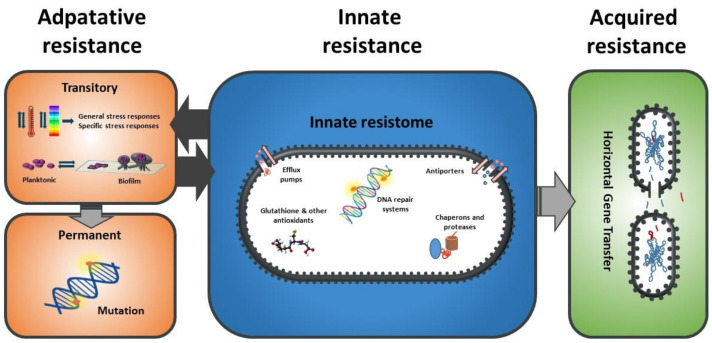
Classification of the mechanisms of stress resistance in bacteria.

As described for antimicrobial resistance, the mechanisms of bacterial resistance to any stress can be classified as innate, adaptive, or acquired [17] (Figure 2). Innate mechanisms include all those structures and potential processes already present and/or active in the bacterial cell when it faces the stressing agent, i.e., they are constitutively and almost continually expressed. Such innate resistance mechanisms include key structures such as the cell envelope robustness, basally expressed homeostatic mechanisms (including membrane pumps, antioxidants, enzymes such as peroxidase, etc.), and damage repair systems (e.g., chaperons and proteases). In the scientific literature, these innate resistance mechanisms are called “classical determinants”. Adaptive resistance includes those genotypic and phenotypic changes arising as a consequence of the exposure of bacterial cells to a given environmental stress. Some examples of adaptive resistance mechanisms include changes in cell permeability, overexpression of protective shock proteins, induction of changes in the targets of stressing agents, and also VBNC, biofilm, and persister development [17,18]. Such changes are generally transient: once the stress ceases, they revert. On certain occasions, however, these phenotypic changes become permanent, for instance, through mutation. Moreover, under stressing conditions, a certain decrease in DNA replication consistency may be essential to produce a more heterogeneous population so that some members possessing the new genes or gene combinations can better survive adverse reigning conditions. This process has been described as adaptive or directed mutation. The mechanisms underlying it include stress-induced errors during DNA synthesis, suppression of normal DNA repair checking and repair mechanisms, transient hypermutability, gene amplification, and stress-induced recombination processes [19]. In addition, bacteria can also increase their resistance to stress through horizontal gene transfer, which can be termed as acquired resistance [18].

Bacterial stress resistance mechanisms can also be classified as specific and/or cross-protective, depending on the range of stressing agents to which the structure or molecule provides protection. In the first case, the triggered response only protects against the same agent that induces it. As described below, general stress response is the major representative of the second type of stress resistance response.

Virulence, on the other hand, can be defined as the ability of a microorganism to cause disease in the host [20]. In this review, we will refer only to humans. The virulence factors of *Salmonella* have been studied extensively, and include flagella, capsules, plasmids, adhesion systems, hemagglutinins, exotoxins, endotoxins, invasins, and type 3 secretion systems (T3SS) encoded on the *Salmonella* pathogenicity islands (SPI)-1 and SPI-2 and other SPIs [21,22,23,24]. The particular role of each of these components, along with their regulation, have likewise been thoroughly investigated, albeit not yet fully elucidated; excellent reviews on the topic can be found elsewhere [25,26,27,28,29]. In order to cause foodborne disease in humans, *Salmonella* cells should survive the (sub)lethal environmental conditions of the gastrointestinal tract (acid, bile salts, low oxygen tension, commensal bacteria, etc.) which are part of the host’s defenses against infection. Thus, a virulent strain or cell should not only be capable of invading intestinal epithelial cells, but also of surviving those harsh conditions. However, in this review we shall distinguish, whenever data are available, between phenomena clearly related to stress resistance (e.g., to the acid pH of the stomach, or to ROS within macrophages) and those associated with the mechanisms of cellular/tissue adhesion, invasion, and proliferation.

Finally, the ability to replicate in a given environment is called growth fitness, or bacterial fitness. As indicated for stress resistance, this definition implies that one should determine the environment in which growth is measured: i.e., a strain might be able to grow faster than other strains under certain conditions, but not under others. In general terms, and for the purpose of this review, we shall consider that a strain has lost growth fitness if acquisition of resistance has resulted in a decrease in its growth rate or in its final concentration in either a standard rich medium (such as TSB, BHI, LB) or in a medium with limited nutrient availability but, in both cases, in which no other stressing agent (such as pH, NaCl concentration, elevated or low temperatures, presence of antimicrobials, etc.) is acting. It is evident that situations such as these, including growth at pH or a_w_ close to the boundaries, are of utmost relevance for food safety, but would make the discussion too complex for the scope of this review and will therefore not be addressed herein.

## 3. *Salmonella* Stress Resistance Mechanisms: Impact on Virulence and Growth Fitness

### 3.1. Non-Specific Stress Responses

#### 3.1.1. The General Stress Response (GSR)

Alternative sigma factors are probably the most relevant strategy developed by bacteria when they face adverse conditions [1]. Among them, alternative sigma factor σ^S^ (also called σ^38^ or RpoS) of RNA polymerase (RNAP) is regarded as the master regulator of the general stress response in many Gram-negative bacteria, including *Salmonella* [30,31,32,33]. Expression of RpoS is induced upon exposure of bacterial cells to various stresses such as acid, heat, and oxidative stress, among others, and also upon entry into stationary phase [34]. Recent results have demonstrated that RpoS directly or indirectly modulates the expression of 38% of the observed *S.* Typhimurium proteome [33].

As might be expected, induction of RpoS leads to the synthesis of a number of proteins with a proven role in *Salmonella* resistance to stress, such as Dps, HPII catalase, OstA, OstB, and several acid shock proteins (ASPs). Dps, the most abundant protein in *S.* Typhimurium during stationary growth phase, is a bacterial ferritin involved in the resistance to oxidative stress [35,36,37]. The *katE* gene, encoding the HPII catalase, is considered to be RpoS-dependent in *Salmonella* [38,39], and it also contributes to the prevention of oxidative stress [40]. RpoS is likewise involved in the transcription of the *otsBA* operon in *S.* Typhimurium [41,42], which plays an important role in countering osmotic stress via regulation of the trehalose synthesis. Under high-osmolarity conditions, trehalose serves as an osmoprotectant [43]. RpoS has also been reported to play an important role in the acquisition of tolerance to organic acid stress by regulating the expression of several acid shock proteins [44].

Nevertheless, it has also been demonstrated that RpoS also controls a broad spectrum of *Salmonella* proteins required for various biological processes, including virulence. According to the data obtained by Rice et al., (2015), RpoS reduces the expression of SPI-1 and SPI-2 at different points in stationary phase cultures [45], which would suggest that RpoS expression might decrease *Salmonella* virulence. However, the transcription SPI-9 operon genes, which contribute to adherence to epithelial cells, increases under low pH and high osmolarity in an RpoS-dependent manner in *S*. Typhi [46] and Nickerson and Curtis observed that a *rpoS* mutant of *S.* Typhimurium exhibited wild-type abilities to attach itself to and invade Int-407 cells and J774 macrophage-like cells [47]. It has also been reported that *rpoS* and RpoS-dependent genes are highly expressed upon entry into macrophages and epithelial cells [38,48], that RpoS induces the expression of SEF14 fimbriae [49], and that RpoS is required for the expression of the plasmid encoded *spv* genes, which, in turn, are required for intracellular growth in deep lymphoid organs such as the spleen and the liver, and which are associated with strains causing non-typhoid bacteremia [50,51,52,53]. These latter results would at least partially explain why deletion of *rpoS* or altered *rpoS* alleles leads to a decrease in *Salmonella* lethality in mice [54], and why *S*. Typhimurium *rpoS* mutants demonstrated a decreased ability to colonize murine Peyer’s patches after oral inoculation as compared to its wild-type virulent parent strain, which indicates that RpoS-dependent gene expression was required for the initial stages of systemic infection [47].

In summary, contradictory observations have been published with regard to the impact of RpoS expression on *Salmonella* virulence and further work would be needed in order to fully elucidate the role of RpoS in *Salmonella* pathogenesis; this role is undoubtedly a highly complex and tightly regulated multi-step process in which attachment and invasion of epithelial cells is as important as survival to acid, bile, oxidative stresses and other host defense mechanisms, and intra and extra cellular proliferation, and consequently, RpoS might be playing different roles in each of these steps.

In spite of the relevance of RpoS, it should be noted that stress-sensitive *rpoS* mutants are surprisingly common among natural isolates of the closely related *E. coli*. A similar phenomenon has been observed for *S.* Typhi, but not for *S*. Typhimurium [55]. Although it has not yet been demonstrated in *Salmonella*, it is assumed that the natural abundance of mutants in the *rpoS* gene would be due to the so-called Growth Advantage in Stationary Phase (GASP) phenotype that these mutations confer (35, 22). Thus, cells with a reduced RpoS activity can grow better in media with low levels of nutrients, and also seem to possess an advantage in competitive colonization of the intestine [56]. This means that a RpoS-related trade-off between survival potential and nutritional competence would exist: a powerful mechanism that gives rise to phenotypic heterogeneity. The reasons why these *rpoS* mutants are not so common in *S.* Typhimurium remain to be elucidated: one reason might be that *S*. Typhimurium uses RpoS for the expression of important virulence genes [55], although, as pointed out above, the role of RpoS on *Salmonella* virulence seems to be very complex. Finally, it should be noted that RpoS also controls a number of genes involved in other highly relevant cellular processes, such as biofilm formation in *S*. Typhimurium [30,57,58].

#### 3.1.2. The Extracytoplasmic Stress Response (ESR)

Besides RpoS, *Salmonella* encodes other alternative sigma factors, such as RpoH (σ^H^) and RpoE (σ^E^). The latter is essential for *Salmonella* survival under conditions of extracytoplasmic stress. Inducers of the RpoE pathway include acid stress, oxidative stress, heat shock, carbon starvation, biofilm formation, ultraviolet A (UV-A) radiation, P22 phage, and hypo-osmotic shock, among others [59,60,61]. Activation of the RpoE pathway is generally due to the accumulation of misfolded and/or mis-translocated outer membrane proteins (OMPs) or lipopolysaccharides (LPS) within the periplasm [60].

The role of RpoE in *Salmonella* pathogenesis has been recently reviewed by Hews et al., (2019) [62]. Early studies of the relationship between RpoE and *Salmonella* virulence already revealed that deletion of RpoE rendered *Salmonella* cells viable but avirulent in a murine infection model: this was initially thought to be mainly related to their increased susceptibility to a variety of stresses [63,64,65]. Nevertheless, recent studies have demonstrated the existence of further links between RpoE and coordination of virulence gene expression. Thus, RpoE would also upregulate SPI-2 genes [66,67], and it has also been observed that deletion of the RpoE-dependent genes *skp*, *surA* and/or *degP* (alone or in combination with other genes) results in a significant loss of virulence [68,69,70]. In addition to these proteins, RpoE-regulated sRNAs have also been linked with virulence [71]. Furthermore, in *S*. Typhi, *rpoE* mutants displayed a reduced expression of the pathogenicity islands SPI-1 and SPI-2 encoding the T3SSs required for invasion and intracellular survival, and, therefore, were attenuated for invasion and intracellular survival [72,73]. It should also be noted that another system regulating the response of *Salmonella* to envelope stress, the Cpx system, has also been proved to be involved in the regulation of virulence in *S.* Typhimurium [74,75].

The relationship between RpoE expression and growth fitness in *Salmonella* has also been investigated. In the study conducted by Shetty et al., (2019), *rpoE S.* Enteritidis mutants grown in Luria-Bertani (LB) broth at room temperature showed an extended lag phase [76]. Similar results have been obtained for *S.* Typhimurium by Testerman et al., (2002) and Humphreys et al., (1999) [63,64]. Supplementation of glucose in LB medium as well as growth in M9-G medium rendered normal growth curves of the *rpoE* mutant. In addition, results obtained in the latter study led to the conclusion that the *rpoE* mutant is not able to properly utilize carbon sources other than glucose, since replacement of glucose with succinate as the carbon source led to a considerably extended lag phase.

### 3.2. Specific Stress Responses

#### 3.2.1. Acid Stress

Food acidification is one of the most widely used methods to control the growth—or accelerate the inactivation—of undesirable microorganisms, including foodborne pathogens such as *Salmonella*. Thus, fermentation, a naturally occurring form of acidification, has long been used for food preservation, as has acidification by direct addition of organic and other appropriate acids [77]. Exposure of *Salmonella* cells to acid conditions leads to the activation of the acid tolerance response (ATR). As described in Álvarez-Ordoñez et al., (2011), the ATR consists of 3 main systems: pH homeostatic systems, modifications of the membrane composition, and synthesis of acid shock proteins (ASPs) [78]. To date, it has been demonstrated that exposure to acidic conditions results in the induction of at least five regulons leading to the synthesis of ASPs: Fur, PhoPQ, OmpR, RpoE, and RpoS [61,78].

The Fur protein controls a subset of ASPs, which contribute to the *S*. Typhimurium exponential-phase ATR, and confer protection mainly against organic acid stress [79,80]. Deletion of *fur* in *Salmonella* attenuates its virulence [54,81,82], and a new role for Fur as a regulator of the expression of the SPI-1 type III secretion system has been described [83], suggesting a role for this regulator in pathogenicity. Induction of PhoPQ and its regulon by acidic conditions (including inside the gastric tract and within macrophage phagosomes) has also been demonstrated [44,80,84,85,86,87]. It should be noted that, as described for Fur, several studies have shown that *Salmonella* strains harboring null alleles of the *phoP* or *phoQ* gene were highly attenuated for virulence [82,88,89,90]. Acid shock also induces OmpR by means of its phosphorylation from the phosphate donor acetyl phosphate. OmpR, in its phosphorylated state, triggers the expression of various genes involved in the acid-inducible stationary-phase ATR [91,92,93]. Interestingly, several studies have connected OmpR with *Salmonella* virulence, mainly through the regulation of the SPI-1- and SPI-2-encoded genes, thereby observing that *ompR* mutants are highly attenuated in mice [82,94,95,96,97]. Finally, both RpoS and RpoE have also been shown to be involved in the ATR of *Salmonella* [61,80,98]. The impact of the induction of both sigma factors on *Salmonella* virulence was discussed above.

Although all these data might suggest that exposure to acid stress would lead to an increase in *Salmonella* virulence, results obtained seem to indicate that not all virulence factors/pathways would be equally affected by acid shocks. On one hand, Ryan and co-workers observed that exposure of *S*. Typhimurium to acid conditions led to a downregulation of some SPI-1 genes [99], and, similarly, Kim et al. [100] found that the *invE* gene (SPI-1) was downregulated in lysogenic *S*. Typhimurium when treated at pH 3.0, 4.0, and 5.0 for a short time. On the other hand, acid shock would lead to an upregulation of SPI-2 genes [99,101]. In addition, differences in these responses have been observed when comparing planktonic and biofilm *Salmonella* cells [102,103]. Such differences between activation of SPI-1 and SPI-2 might be explained by the fact that SPI-2 plays a key role in *Salmonella* intracellular pathogenicity where acidification of the phagosome serves as a signal for SPI-2 induction. By contrast, as indicated by Kitamoto et al., (2016), virulence genes will not be required in other acidic environments, such as the stomach, which might explain why other SPI-1’s would be repressed upon exposure to acid conditions [104].

In any case, these results and conclusions were all obtained under conditions leading to a transient (acid) stress response; they are therefore not so relevant from a food safety perspective, at least if we assume that all the cells suffer such acid shock in the stomach. The results obtained by Karatzas et al., (2008) indicated that sustainable *Salmonella enterica* acid-resistant variants, obtained upon repeated cycles of acid challenge and growth, displayed an increased expression of SEF17 fimbriae and a reduced virulence [105]. Nevertheless, since these variants’ mechanisms of acid adaptation are unknown, it is very difficult to compare these data with the data previously presented. In Table 1, some examples of stable *Salmonella* variants obtained after successive exposure to different selecting agents are listed. The impact of the resistance responses developed on the virulence and growth fitness of these strains is also included.

On the other hand, Karatzas et al., (2008) observed that the acquisition of acid resistance did have a fitness cost (at neutral pH) for *Salmonella* cells [105]. Apart from the overexpression of ASP and of pH homeostasis systems, it is plausible that an increase in the amount of Cyclopropane Fatty Acids (CFA) caused by adaptation to acid shock might also impose some fitness cost, since they contribute to membrane rigidification, and, whereas rigid membranes seem to be associated with stress resistance, exponential growth phase cells tend to display membranes that are more fluid, which is normally related to active growing [106,107].

#### 3.2.2. Osmotic Stress

Hyperosmolarity and desiccation—usually in combination with other agents—are frequently used by the food processing industry as a means to prevent bacterial proliferation, in food products [108]. It is remarkable that although *Salmonella* is not able to grow at water activities below 0.93, the presence of *Salmonella* has been reported in numerous low-moisture foods [108].

The mechanisms of *Salmonella* survival in low a_w_ environments and the regulation thereof are partially but still not completely understood. Thus, when *Salmonella* cells are exposed to a high osmotic pressure environment, they develop various adaptive responses that include the accumulation of solutes such as K^+^ and osmoprotectants (betaine, proline and trehalose), changes in the composition and permeability of their membranes, degradation of ribosomal RNA molecules, and many other consequences of the activation of both RpoS and RpoE [78,108,109,110].

The transcriptome of *Salmonella* cells after their exposure to low a_w_ environments has been studied by various researchers [110,111,112,113,114]. In some cases, exposure to low a_w_ can even provoke *Salmonella* cells to enter into a physiologically dormant state [111]. In general, however, in spite of the different conditions assayed, almost no gene related to virulence was found to be up- or down-regulated in these studies. By contrast, Kröger et al., (2013) observed that exposure of *S*. Typhimurium cells to a 10 min 0.3 M NaCl shock resulted in an increased expression of several SPI-2 genes and of some effector proteins such as SseK1, AvrA, GtgE or SptP [115]; Huang et al., (2007) observed that a 120 min 0.3 M NaCl shock resulted in an increased expression of SPI-1 genes, although in their case they worked with *S*. Typhi cells [113]. Nevertheless, it should be noted these conditions are not comparable to those applied in the previously indicated studies.

These results, especially from authors who did not observe any change in the expression of virulence factors, contrast with investigations that studied the effect of osmotic shocks/desiccation on the capability of *Salmonella* cells to invade CaCo2 cells, such as the study by Lang et al., (2017), who observed that drying increased the invasion capacity of *S*. Typhimurium and *S*. Senftenberg cells [116], and the study by Yoon et al., (2013), who observed that exposure of *S*. Enteritidis cells to NaCl increased their cell invasion efficiency (Table 1) [117].

These later results might explain why salmonellosis, epidemiologically linked to the ingestion of a contaminated low a_w_ product, may arise from a low infectious dose (of the order 10–100 CFU) in contrast with the infectious dose following ingestion of other contaminated foods (>10^5^ CFU) [118]. Nevertheless, it cannot be discarded that other factors such as an underestimation of actual numbers of bacteria contaminating the low a_w_ food matrix (because of clumping, filamentation, or entry into VNBC state) or an increase in stress resistance of *Salmonella* cells (due to the development of cross-resistance mechanisms because of low a_w_ matrixes that could be exerting a protective effect) could also be contributing to this phenomenon.

#### 3.2.3. Oxidative Stress

In order to reduce the total microbial load in surfaces, the food industry often uses disinfectants such as sodium hypochlorite or hydrogen peroxide. Nitrites and nitrates that lead to the formation of Reactive Nitrogen Species (RNS) are also found in some food products and/or are intentionally added to prevent microbial growth. These are nevertheless not the only sources of oxidative damage that *Salmonella* cells can encounter in the course of the food processing chain, since it has been already demonstrated that many agents/technologies lead to the generation of Reactive Oxygen Species (ROS) within microbial cells [119].

Once inside the host, *Salmonella* cells are confronted with professional phagocytic cells, eventually also leading to exposure to ROS and RNS [120]. Furthermore, ROS are formed by bacterial cells themselves through the activity of the respiratory electron transport chain [121]; *Salmonella* has therefore developed a wide range of mechanisms to cope with them. They include the synthesis of enzymes capable of detoxifying them, such as superoxidodismutases, alkyl hydroperoxide reductases, catalases, peroxidases, thioredoxin, periplasmic oxidoreductases, DNA and protein damage repair enzymes (such as RecA, LexA, and SulA), and various chaperons and proteases. Further mechanisms include reductants such as glutathione, but also the induction of changes in membrane permeability as described by van der Heijden et al., (2016) [122]. Furthermore, it has been demonstrated that *Salmonella* induces and makes use of an inflammatory response with its accompanying ROS production, since the latter provides *Salmonella* with a competitive advantage over the gut microbiota [123,124,125].

The intimate relationship between some of these defense mechanisms and bacterial virulence is well illustrated by the fact that periplasmic superoxide dismutase SodCI becomes upregulated even when *S*. Typhimurium replicates in non-activated murine monocytic cells. One should thereby bear in mind that periplasmic superoxide dismutase SodCI is likewise part of the *Salmonella* PhoP/PhoQ virulence regulon, which also includes the SPI-1, SPI-2, and the *spv* genes [126,127,128]. However, it should also be noted that the regulation of the oxidative stress response is very complex: a number of regulators are involved, and some of them induce opposite effects on *Salmonella* virulence. Thus, in addition to RpoS and RpoE, the SoxR/SoxS and the OxyR regulons have been shown to play a major role in *Salmonella* response to oxidative stress [62,129]. Nevertheless, neither the SoxR/SoxS nor the OxyR regulon seem to play a relevant role in *Salmonella* pathogenesis [109,130] although the latter seems to contribute to intestinal colonization and is a target for the immune system [131,132]. *Salmonella* NsrR regulon is activated as a consequence of nitrosative stress, and deletion of some of its genes does result in a reduced virulence in mice; further work shall be required, however, to elucidate whether this regulon plays a role in processes other than stress resistance [133].

Transcriptomic analysis of *Salmonella* responses to chlorine, hydrogen peroxide, and nitric oxide revealed that exposure to chlorine led to a downregulation of SPI-1-regulated genes in *S*. Typhimurium LT4, although almost no differences in the level of expression of these genes were observed between control and chlorine-treated for *S. Enteritidis* LT2 cells [134]. In a similar study, Cadena and co-workers [135] observed that exposure of *S*. Heidelberg to acidified calcium hypochlorite or peroxyacetic acid, only resulted in minor changes in the expression of virulence related genes. On the other hand, Kröger et al., (2013) reported that peroxide and nitric oxide shocks resulted in downregulation of the SPI-2 genes; however, it should be noted that the control cells in their study (non-oxidative stressed cells) were grown in an acidic phosphate-limiting minimal medium specifically designed to induce SPI-2 transcription [115].

To the best of our knowledge, isolation of mutants/clones with an increased resistance to oxidative stresses has only been reported in the works by Karatzas et al. [136,137]. Their results showed that *S.* Typhimurium variants with an increased resistance to an oxidizing compound blend displayed a decreased ability to invade CaCo-2 cells and also lower growth rate and yield.

#### 3.2.4. Starvation

Starvation or nutrient limitation is, paradoxically, quite common along the food processing chain. Thus, bacteria can be under starvation when they are located in food processing equipment or surfaces, but also on the surface of many products (e.g., those possessing a shell) or even inside certain products (e.g., iron availability is limited in egg white). The starvation stress response (SSR) of *S.* Typhimurium encompasses the genetic and physiological changes that occur when they are starved of an essential nutrient, e.g., phosphate (P), carbon (C), or nitrogen (N) [138]. Depending on the limiting nutrient, however, the changes induced by the cell will differ, although certain common regulons are induced by the absence of almost any of them.

The starvation stress response (SSR) is regulated by different proteins/systems including RelA (responsible for the synthesis of guanosine tetraphosphate (ppGpp)), SpoT (a bifunctional synthetase and hydrolase of ppGpp), Crp (the cyclic AMP (cAMP) receptor protein), CsrA (a carbon-storage regulator) [139], and DksA (an RNA polymerase-binding protein) [140,141,142,143,144]. RpoS is also induced by a variety of starvation conditions whereas RpoE has been proven to be induced by carbon starvation [138,145].

Regarding the link between the SSR and *Salmonella* virulence, it has been demonstrated that *Salmonella* cells deficient in ppGpp synthesis display a reduced expression of *hilA* and *invF*, encoding major transcriptional activators required for SPI-1 gene expression, and that they are non-invasive in vitro and highly attenuated in vivo [146]. (p)ppGpp is also required for the activation of SPI-2 gene transcription [147]. Furthermore, it was very recently demonstrated that an *S*. Typhimurium mutant with a SpoT variant without hydrolase activity displayed a decreased expression of SPI-2 genes [143], indicating that RelA and SpoT would play different roles in *Salmonella* pathogenesis. On the other hand, CsrA is a post-transcriptional regulator that controls the expression of SPI-1 and SPI-2 through direct repression of *hilD* translation initiation during growth in rich media [139,148,149]. End products of metabolism and amino acid starvation stimulate *csrB/C* transcription of two inhibitory small RNAs that sequester CsrA and, thus, inhibit its repression of *hilD.* Finally, deletion of *crp* has been proven to reduce *Salmonella* pathogenicity of different serovars [150,151,152,153] and the deletion of DksA led to a decrease in the virulence of *S.* Typhimurium in mice [154]. The results of Kroger et al., (2013) are thereby consistent with the hypothesis that starvation causes significant changes in *Salmonella* virulence, since they observed that growth in PCN (a minimal medium) resulted in an increased transcription of many SPI-1 genes, as compared to growth in LB [115].

Iron, on the other hand, is not only an essential growth factor for *Salmonella*, but also seems to play a very relevant role in its pathogenicity. Thus, the ability to acquire iron has been suggested as a key factor that determines the ability of *Salmonella* cells to outcompete the commensal microbiota within the gut; it also serves as a signaling element that regulates various genes, including virulence-associated genes [155]. The amount of available iron also seems to determine the adhesion and invasion capability of *Salmonella* cells [156,157,158]. Starvation for iron occurs within the food chain under conditions such as those described above, but also in some food products such as egg white, which is a very relevant matrix for salmonellosis [159].

The master regulator Fur (ferric uptake regulator) is the main transcription regulator in *Salmonella* in response to iron availability [160,161]. Thus, transcriptional repression by Fur is relieved when iron is limited, leading to the expression of iron acquisition systems, including those that produce and secrete iron-chelating siderophores such as enterobactin and salmochelin [162]. A clear example of the link between Fe metabolism and virulence is the fact that deletion of genes of these iron uptake systems (such as *tonB*) resulted in *Salmonella* with a lower ability to invade Caco-2 [158]. Transcriptomic analysis has revealed that exposure to low Fe concentrations leads to the induction of the *sitABCD* iron transporter located in the SPI-1 [115]. Although no effect of the expression of SPI-2 genes was observed upon exposure to low Fe concentrations in that study, in other studies increased expression of SPI-2-associated virulence genes upon exposure of *Salmonella* cells to these conditions has been reported [155,163]. Finally, Dostal et al., (2014) observed that the number of *Salmonella* cells capable of invading a CaCo2-HT29-MTX co-culture was greater when the media had low iron concentrations [156]. In other studies, however, it has been observed that increasing the iron concentration has the same effect [158].

#### 3.2.5. Modified Atmospheres

Modified-atmosphere technologies are capable of extending the shelf life of food products by minimizing the physiological, chemical, and microbial decomposition of foods in an atmosphere that differs from the normal composition of air [164]. For this reason, such technologies, especially Modified Atmosphere Packaging (MAP), are extensively used nowadays for preserving fresh, minimally processed, and processed food products.

*S. enterica* is a facultative anaerobe; it thrives in an anaerobic environment by performing fermentations and/or by using alternative electron acceptors, such as nitrate or fumarate [165]. FNR (also called OxrA) is considered to be the main regulator of the adaptive response of *S. enterica* to lack of oxygen, and is a cytoplasmic oxygen sensor that can bind promoter sequences. Upon interaction with the RpoA subunit of RNA, it induces the transcription of a variety of genes required for anaerobic metabolism, whereas it represses many of the genes encoding enzymes involved in aerobic electron transport, oxidative phosphorylation, and some tricarboxylic cycle enzymes [78]. On the other hand, ArcAB is a two-component signal transduction system induced under microaerobic and anaerobic conditions that suppresses the expression of genes encoding enzymes of the tricarboxylic cycle, leading to a decrease in the amount of generated ROS [166].

Apart from their role in regulating the main metabolic and energy pathways of *Salmonella*, both regulators have been shown to affect the expression of virulence factors. Thus, OxrA/FNR regulates numerous virulence genes within *Salmonella* pathogenicity island 1 (SPI-1), the virulence operon *srfABC*, and also some flagellar genes (*mcpAC*, *cheV*). Furthermore, an *oxrA* mutant was shown to be non-motile and attenuated in vivo [167]. ArcA also induces certain virulence genes but represses others. This fact, together with the low number of genes it regulates and the complexity of their regulation, would explain why an *arcA* mutant showed virulence defect in mice [168,169]. Kroger et al., (2013) also observed an increased expression of SPI-1 genes when *S*. Typhimurium was grown in anaerobiosis as compared to aerobic conditions; not, however, after a 30-minute anaerobic shock [115]. Furthermore, the expression of SPI-1 genes increased even further when anaerobically grown cells were exposed to a 30-minute aerobic shock, which suggests that the relationship between the composition of the atmosphere and the expression of SPI-1 factors would be more complex than was initially expected. In any case, various studies have observed that *Salmonella* cells grown in anaerobiosis do display an increased adhesion and invasion capacity of cultured cells [170,171,172,173].

#### 3.2.6. Chemical Stressors: Detergents and Disinfectants

Bile salts, which are released after food intake from the gallbladder into the duodenum, act as detergents and, therefore, exert their effects on bacterial cell membranes; they can also have numerous other effects on further molecules such as RNA, DNA, and proteins [174,175,176]. Although they are not relevant in terms of food preservation, the response of *Salmonella* to bile salts has been widely studied for obvious reasons, and can also serve as a model for the responses that other detergents can trigger.

The deletion of various proteins involved in *Salmonella* bile resistance such as AcrB, Dam, PhoPQ, and Wec has been proven to reduce the virulence of *Salmonella* cells in in BALB/c mice [88,177,178,179,180]; this seems to be more likely, however, due to the decrease in stress (bile) resistance than to a decrease in the expression of virulence factors. Prouty and Gunn (2000) demonstrated that *S.* Typhimurium grown in the presence of bile is able to invade epithelial cells at only 4% of the level of cells grown in the absence of bile, and transcription of invasion gene regulators (*sirC* and *invF*) was shown to be repressed in the presence of bile, resulting in decreased transcription of SPI-1 genes [181,182]. Recently, Urdaneta et al., 2019 observed that 7 out of 10 *Salmonella* bile-resistant mutants recovered from the gallbladder of infected mice showed one or more virulence-related defects; they suggested that resistance to bile would be achieved at the expense of virulence impairment, and that it may involve fitness tradeoffs in certain cases [183]. Nonetheless, most authors agree that *Salmonella* may use bile as an environmental signal to repress its invasive capacity in the intestinal lumen, where bile concentrations are high, and invasion may then be initiated after transiting the mucus layer [104,181,182].

Disinfectants can be broadly grouped into oxidizing agents, surface active compounds, and iodophores [184]. Widely used sanitizers, such as halogen-based compounds, peracetic acid (PAA), ozone, and hydrogen peroxide, fall within the group of oxidizing agents; their impact on *Salmonella* virulence and growth fitness was discussed above. However, surface-active compounds, such as acid anionic compounds and quaternary ammonium compounds (QACs), are also frequently used in food industries. In a recent study by Cadena et al., (2019), the authors observed that the effect of exposing *S.* Heidelberg cells to cetylpyridinium chloride on the transcription of virulence factors depended on the strains studied [135]. Thus, whereas for one of the strains the upregulation of some SPI-1 genes was observed, for the other one no differences were observed. By contrast, Kautz et al., (2013) observed that *S.* Enteritidis strains with reduced susceptibility to dodecyltrimethylammonium chloride were found to be less invasive (Caco-2 cells were used) and had fewer fimbriae, and that the majority had lower expression levels of *fimA*, *csgG*, and *spvR* (3 out of 4) than those of the parental strain, thereby suggesting a generally decreased pathogenicity (Table 1) [185]. Similar results to those of Kautz and co-workers were obtained by Karatzas et al., (2007), who observed that stable variants of *S.* Typhimurium SL1344 exposed to three different commercial disinfectants displayed a decreased invasiveness of Caco-2 cells (Table 1) [136,137]. These authors also observed that acquisition of resistance to these disinfectants also resulted in a decreased growth fitness in LB broth.

#### 3.2.7. Chilling

Although *Salmonella* is a mesophilic microorganism unable to grow below aprox. 7 °C, *Salmonella* cells are often exposed to refrigeration and freezing temperatures within the food chain. The adaptation mechanisms of *Salmonella* cells to cold have been recently reviewed by Ricke et al., (2018), and include changes in the composition of their envelopes (i.e., membrane fluidification), the induction of the synthesis of a series of proteins (Cold Induced, Cold Shock and Cold Acclimatization Proteins), and changes in DNA supercoiling [186].

Regarding the impact of the cold stress response on *Salmonella* virulence, it should be noted that the NusA protein, which has been identified as induced under cold temperature conditions [187,188], was found to impact *hilA* expression, the transcriptional activator of *Salmonella* Pathogenicity Island-1 [189].

On the other hand, whereas Kröger et al., (2013) did not observe any significant change in the expression of SPI-1 and SPI-2 genes after a 10-minute shock at 10 °C [115], Shah et al., (2014) observed that exposure of *S*. Typhimurium cells to a temperature of 5 °C for 48 h resulted in induction of several groups of virulence genes, including T3SS-associated genes located on SPI-1 and SPI-2 [190]. The latter authors also studied the effect of this cold shock on the ability of *Salmonella* to adhere to and invade Caco-2 cells, and observed an increase in both phenomena.

#### 3.2.8. Heat

Heat has been widely used in the food industry as a preservation agent since it is capable of inactivating most microorganisms and enzymes present in foods. Therefore, heat is a method capable of simultaneously guaranteeing food safety and food stability [191]. Given its relevance, the mechanisms of microbial inactivation by heat and the stress responses that bacteria can develop upon exposure to sublethal heat treatments (the heat shock response) have been extensively studied. The heat shock response in *Salmonella* involves activation of various regulons including those controlled by the sigma factors RpoS, RpoE, and RpoH [192]. The role of RpoS and RpoE in *Salmonella* stress resistance, pathogenesis, and growth fitness was discussed above and, therefore, will not be reviewed in this section. On the other hand, the sigma factor σ^32/H^ controls more than 30 proteins, most of which are associated chaperones and proteases [31,193,194,195,196,197]. Although both RpoH and RpoE are alternative sigma factors, RpoH regulates Heat Shock Proteins (HSPs) for the cytoplasmic components, whereas RpoE regulates the extracytoplasmic (cell envelope) proteins in response to high temperatures, as it does with other envelope stress factors [60,198,199,200,201,202,203]. As described for *rpoS, rpoH* gene expression is also regulated by RpoE [204,205,206,207,208,209].

Increased expression of virulence genes in *Salmonella* upon exposure to sublethal heat shocks or growth at high temperatures has been reported in a number of studies such as that of Yang et al., (2013), who observed that virulence-related genes—*spvR*, *hilA*, *avrA*— were more expressed in *S*. Enteritidis cells the higher the growth temperature (between 10 and 42 °C), while *sefA* maximum expression was observed when cells were grown at 37 °C [210]. Recently, Dawoud et al., (2017) reviewed the potential relationship between heat resistance (or the heat shock response) and virulence [211]. In their review, they indicated that various *Salmonella* heat-shock proteins have been shown to play a role in pathogenesis, including FtsH, FkpA, SurA [68,69,212,213,214,215,216]. Similarly, it has been demonstrated that increasing growth temperature to at least 37 °C leads to a decrease in the binding capacity of H-NS to DNA (AT-rich sequence), which leads, in turn, to virulence gene (SPI-1, 2, 3 and 5) expression [217,218,219,220,221,222,223,224]. The same authors also point out that exposure of *Salmonella* cells to sublethal heating conditions might also exert an influence on their virulence via changes in DNA topology, such as a DNA supercoiling which would lead, in turn, to SPI-1 gene expression [225,226] or could be due to the generation of bends in the AT-rich sequence regions situated in the 5′-end upstream of the promoter region that would influence the interaction between RNA polymerase and the promoter region, thereby altering gene expression [227,228]. Information concerning the impact of other heat resistance mechanisms, such as membrane rigidification, on *Salmonella* virulence is very scarce; further research is necessary in this domain.

In parallel, various investigations have studied the influence of the development of resistance to heat of *Salmonella* cells on its virulence by using different models. Sirsat et al., (2001) reported that exposing *Salmonella* cells to a heat shock of 42 °C resulted in an improved adhesion to Caco-2 cells, but not in a higher degree of invasion [192]. The same authors reported that upon that heat shock the genes of two *Salmonella* pathogenicity islands (SPI-2 and SPI-5) were upregulated, which would explain their higher adhesion and would confer a higher chance of survival in the host while genes of SPI-1 were downregulated. In another study, Lang et al., 2017 compared the invasion ability of dried *Salmonella* cells after different heat treatments and likewise did not find any differences with the non-heat treated cells, although, in this case, the reduced metabolic activity of dried cells might explain the absence of effects of the heat treatment [116]. To the best of our knowledge there is only one work in which the virulence of stable variants/clones of *Salmonella* cells has been examined and the results of this work indicated that the heat resistant strain displayed a lower pathogenicity after intraperitoneal injections into 20–22 g mice [229].

To sum up, and although the relationship between the heat shock response and virulence in *Salmonella* seems to clearly exist, further work is still required to explain certain (at least apparently) contradictory findings, such as the diverging results obtained regarding expression of SPI-1 genes upon exposure to sublethal heat conditions or why the already demonstrated overexpression of virulence related genes after a heat shock is not directly translated into an increased invasivity or virulence in animal models.

On the other hand, the development of heat resistance would probably have a negative impact on *Salmonella* growth fitness, always considering non stressing conditions. This assumption is based on the fact that, in *Salmonella*’s close relative *E. coli*, overexpression of GroEL/GroES is required in order to enable growth at high temperatures (48.5 °C) [230]. It has nevertheless also been demonstrated that overexpressing GroES imposes a high fitness cost [231]. Similarly, Ezemaduka et al., (2014) observed that expression of a protein from *Caenorhabditis elegans* allowed *E*. *coli* cells to grow under temperatures up to 50 ºC [232]. Authors have indicated that this protein would help to maintain cell envelope integrity. However, an excessive rigidification of the membrane would also have a negative impact on growth rates unless other compensatory mechanisms were activated [233].

#### 3.2.9. Non-Thermal Technologies

Given the limitations of heat treatments for the preservation of food quality while ensuring food safety, a considerable number of so-called “Non-thermal technologies” have been investigated as alternatives to thermal treatments, with the objective of meeting the required safety or shelf-life demands while minimizing eventual effects on nutritional and quality attributes. However, the amount of information regarding the impact of novel non-thermal technologies on *Salmonella* virulence and growth fitness is quite scarce. This is not surprising, since even the amount of studies that deal with the development of microbial homologous adaptation to technologies such as Cold Atmospheric Plasma, Pulsed Electric Fields, Ultrasound, High Hydrostatic Pressure, and UV light is very limited and, to the best of our knowledge, nonexistent for *Salmonella* except for studies by Maâlej et al., (2014) and Timmons (2018) [234,235].

Some works dealing with the development of resistance to ionizing radiation in *Salmonella* cultures and its impact on other aspects of its physiology were carried out in the late 1960s and early 1970s. Most of the data obtained, included in Table 1, indicate that the increase in resistance to ionizing radiation acquired did not result in major changes in either the growth fitness or the pathogenicity of *Salmonella cells* [229,236,237,238,239], although in some works a decreased growth ability was found in minimal medium [236] or at relatively low temperatures [237,238]. Also a decrease in pathogenicity *S*. Typhimurium cells with repeated exposure to ionizing radiation was observed by Previte et al., (1971) [239].

Regarding resistance to UV light, Maalej, et al., (2014) observed that pre-adaptation of three different *Salmonella* strains led to an increase in the proportion of CFA in their membranes [234], thus consistent with the results obtained by Gayán et al., (2013), who suggested that membrane fluidization would lead to an increased sensitivity to UV in *E. coli* [240]. As described for acid stress, membrane rigidification might impose a fitness cost unless compensatory changes were set up. Since *rpoS* as well as many genes related to the oxidative stress response have been shown to be related to *Salmonella* UV resistance [241], it is plausible that the development of UV resistance might have the same effects, at least to some extent, on virulence and growth fitness as those discussed above. This nevertheless remains to be demonstrated.

Information regarding the impact of the development of resistance to Pulsed Electric Fields (PEF) on *Salmonella* virulence and growth fitness is likewise scarce, but data obtained to date seem to indicate that development of resistance to PEF after several cycles of treatment and growth of survivors would not result in decreased growth fitness [242]. On the other hand, the results obtained by Sanz-Puig and coworkers (2019) indicate that the development of PEF resistance would result in a decreased virulence of *Salmonella* cells in a *Caenorhabditis elegans* model [243,244]. It should also be noted that a potential link between RpoS activity and PEF resistance was suggested by Sagarzazu and coworkers, which would imply that, although still not demonstrated, an increase in PEF resistance might indeed have a fitness cost and imply changes in *Salmonella* virulence [242]. Sanz-Puig et al., (2019) also studied the impact of the development of HHP resistance after repeated exposure to sublethal HHP treatments on *Salmonella* virulence, and observed, as described above for PEF, that this stress resistance response also led to a decrease in virulence in *Caenorhabditis elegans* [244].

Finally, results obtained by Timmons (2018) indicate that exposure of *Salmonella* cells to Cold Atmospheric Plasma (CAP) might lead to the upregulation of SPI-2 genes, although results were inconclusive due to inconsistency between experimental replicates [235]. The same author also observed that even though *Salmonella* cells were not able to increase their resistance to CAP upon five successive treatments with that technology, transcriptomic analysis revealed an upregulation of SPI-2 genes [235].

### 3.3. Other Stress Responses

#### 3.3.1. Viable but Non-Culturable (VBNC) and Persister Cells

Under some circumstances, exposure to stresses of different types can lead to the induction of the VBNC state in bacterial cells. This phenomenon has also been described for *Salmonella*: although cells maintain some metabolic activity, they can only be cultured (multiply) after a resuscitation step. The stressing conditions that have been shown to induce the VBNC state in *Salmonella* include UV light, osmotic stress, nutrient starvation, and heat, among others [245,246,247]. Recent results have demonstrated that VBNC cells can arise without the input of an obvious stress, similarly to persisters (see below) [248]. The pathogenicity of these cells is still regarded as controversial: this is of major relevance because it raises uncertainty regarding the health risk posed by such VBNC forms. Thus, although some studies observed that microbial cells can retain some pathogenic effects [249,250,251], most studies carried out with *Salmonella* cells have shown a simultaneous loss of culturability and pathogenicity [246,247]. Furthermore, it has been hypothesized that the pathogenicity observed in the former studies might be linked to the reversion of cells to a culturable state [252,253]. Further work will be required to fully elucidate this point: in any case, however, it seems clear that the pathogenicity of these cells would be lower than that of the culturable ones, indicating that the major risk associated with VBNC cells would be the difficulty of their detection. Therefore, the existence of this phenomenon (the VBNC state) really calls into question the suitability of traditional microbiological techniques for enumerating pathogenic microorganisms in foods, along with the validity of risk assessments performed on the basis of these data. On the other hand, one of the major characteristics of VBNC cells is that they require a resuscitation step before they are able to multiply. Thus, although once resuscitated they would display a growth rate similar to non-VBNC cells, the resuscitation step would, in many cases, hamper their multiplication in food products or, at least, delay it significantly.

During growth, genetically clonal bacterial populations contain a small fraction of non-growing, non-dividing cells that arise from transient, reversible phenotype switching. These growth-arrested cells are usually tolerant to antibiotics and are called (antibiotic) persisters [254]. However, it is now known that cells can be induced to become persisters through exposure to stressful conditions, similarly to the VBNC state [254]. The distinction between persisters and VBNC is quite complex, and nowadays it is considered that both survival strategies would represent a continuum between actively growing and dead cells, with VBNC cells being in a deeper state of dormancy than persister cells. Moreover, both phenomena can coexist within the same bacterial population. For a detailed review of the similarities and differences between them, we recommend Ayrapetyan et al., (2018) [248]. Persister cells are thought to be ubiquitous among bacterial species, and have been already described in *S.* Typhimurium [254,255,256]. It is very important to point out that, in contrast to resistant cells, persisters are genetically identical to susceptible bacteria, thereby constituting phenotypic variants of the wild type. They can be formed in different ways, but, upon removal of the stressing agent, they switch back to a normally growing state [257]. To date, the study of this phenomenon has been almost exclusively restricted to the field of clinical microbiology; however, antimicrobial agents commonly used in the food industry, such as nisin, can also induce their formation [258]. It is thus plausible that these cells could also appear in response to other agents. Further work will be required to determine the relevance of this phenomenon from a food safety perspective (apart from resistance to antibiotics); as pointed out above, although persisters are non-growing cells, this is a transitory state with, predictably, no fitness cost apart from the time required to exit from dormancy, which, moreover, is not as deep as that of VBNC cells. Finally, it should be noted that some microbial persisters (*Porphyromonas gingivalis*) would apparently maintain their ability to invade epithelial cells [259].

#### 3.3.2. Biofilms

Biofilms are defined as a mode of growth where cells aggregate and become embedded in a self-produced extracellular matrix, usually in contact with a physical surface. The exact reasons why bacteria aggregate together are not fully understood, but it has been clearly demonstrated that bacteria in the biofilm are resistant to disinfectants as well as to chemical, physical, and mechanical stresses [260,261,262]. The major constituents of *Salmonella* biofilms and the complex network regulating their formation have been described elsewhere [263,264,265]. Although it has been proposed that aggregation could provide *Salmonella* with a mechanism for surviving the harsh conditions of the host intestinal tract to ensure that a “viable and sufficient” inoculum could reach the epithelial layer and, therefore, a lower number of biofilm cells would be required to cause disease, it has been demonstrated that biofilm cells and/or the presence of extracellular matrix factors reduce *Salmonella* virulence in the mouse model of infection [266,267,268]. In line with these results, Mackenzie et al., (2015) observed that expression of SPI-1 was lower in biofilm than in planktonic *Salmonella* cells, although a direct link between the biofilm regulator CsgD and SPI-1 expression has not yet been established [267]. On the other hand, it should be noted that curli and cellulose (components of the *Salmonella* biofilm extracellular matrix) may also play a highly relevant role in host–pathogen interactions [269]. However, deletion of *csgBA* (formerly *agfBA*), encoding the main curli subunit proteins, caused no noticeable impairment of *Salmonella* virulence [270], and various studies seem to indicate that cellulose would hamper *Salmonella* virulence (summarized in Mackenzie et al., 2019) [269]. Several questions nevertheless remain to be solved in order to elucidate the precise role of curli and cellulose in *Salmonella* pathogenesis and the relevance of biofilm formation in this phenomenon.

Some studies have compared the physiological status of biofilm and planktonic cells by determining their growth rates, with contradictory results. Thus, some investigators [271,272] have reported increased biofilm growth rates in comparison to planktonic growth rates, while others [273] have reported the opposite. Further research is still required in order to clarify this point [274,275].

## 4. Impact of Stress Resistance Responses on Other Aspects of Salmonella Physiology

Although, as pointed out above, flagella facilitate adherence to surfaces including the host epithelium, their major function is to enable bacteria such as *Salmonella* to swim through liquid environments and on surfaces, thereby enabling them to chemotactically swim towards nutrients or away from harmful substances [276]. Although it can be expected that their synthesis might be somehow related to the stress resistance responses, the relationship between them is not fully understood in *Salmonella* cells. Thus, whereas in *E. coli* K12 it is well established that RpoS downregulates the expression of flagella, results from Lévi-Meyrueis et al., (2014) indicate that the flagellin genes *fliC*, and to a lesser extent *fljB*, were positively controlled by RpoS, resulting in a decreased motility in a rpoS deletion mutant, even though transcription of the *flhDC* genes encoding the master regulator of flagellar synthesis were slightly upregulated in a *Salmonella ΔrpoS* strain [42]. These authors nevertheless also indicated that, given the complexity of regulatory controls affecting motility, it cannot be determined whether the positive regulation of *fliC* accounts for the effect of RpoS on motility, or if RpoS would be acting by other means as well. On the other hand, RpoE seems to be involved in the downregulation of *Salmonella* motility, and it has been suggested that this downregulation of flagellar synthesis might be helpful in host immune evasion, thus increasing bacterial fitness during infection [277]. Likewise, Ryan et al., (2015) observed that genes belonging to the flagellar assembly and chemotaxis modules, as well as FliA, were highly downregulated under the acid tolerance response of *S*. Typhimurium [99]: the results obtained by Sirsat et al., (2011 and 2015) indicate that heat shock would cause a similar effect. By contrast, exposure to a_w_ 0.11 resulted in an up to threefold increase in the expression of certain genes involved in the biosynthesis of flagella in *S.* Typhimurium cells [278] and the results of Walker and co-workers [279] indicated that particularly surface growth at low pH values, would induce a “hyper-flagellate” phenotype.

Quorum Sensing (QS) regulates numerous important cell functions in both Gram-positive and Gram-negative bacteria, including metabolism, protein synthesis, expression of virulence factors, antibiotic resistance, biofilm formation, biofilm maintenance and dispersal, and entry to stationary phase. It has long been established that in *Salmonella* the production autoinducer 2 (AI-2) (one of the two major bacterial QS systems) is induced by a series of stimuli such as low pH and high osmolarity [280,281]. In addition, a very interesting phenomenon is the ability of CAP to directly disrupt quorum sensing molecules utilized by Gram-negative bacteria (acylhomoserine lactones) [282], which would result in a decreased virulence of quorum-sensing-controlled virulence factors as described for Pseudomonas by Ziuzina et al., (2015) [283].

Finally, the potential impact of the development of stress resistance response to agents and/or technologies commonly used/encountered in the food chain on antimicrobial resistance remains largely unexplored. Given the fact that, in many cases, they share resistance mechanisms (e.g., membrane stability/permeability) it can be expected that a potential link between them can exist. This was already demonstrated by McMahon et al., (2007), who observed that incubation at sublethal high temperature (45 °C) decreased *Salmonella* antibioresistance but by contrast, osmotic (>4.5 NaCl %) and acid (<5.0) shocks resulted in an increase in *Salmonella* resistance to certain antimicrobials [16]. Similarly, Álvarez-Molina et al., (2020) reported that repeated exposure of different microorganisms (including *Salmonella*) to UV-C and CAP led to an increase in resistance to certain antimicrobials, a phenomenon that was linked, depending on the case, to changes in antibiotic cellular targets, to membrane transporters probably involved in the nonselective efflux of antibiotics and, very interestingly, to stress response regulators (Table 1) [284].

## 5. Variability among Salmonella Strains and Serovars

The geno- and pheno-typic diversity of the *Salmonella* genus is not only a well-known fact but also one of its more characteristic features [285]. Many works have studied in different depths the variability in stress resistance among *Salmonella* strains and/or serovars [286,287,288,289,290,291,292,293,294,295]. Nevertheless, the information regarding the ability of the different strains/serovars to develop stress resistance responses (either transient or permanent) is much more scarce. In any case, if as described above, the development of stress resistance can have a cost in terms of growth fitness and/or virulence, it would be reasonable to think that strains and/or serovars that had evolutionarily acquired resistance to particular stresses or conditions, will also probably have lower growth fitness and that, at least, will display an altered virulence ability. Similarly, other phenotypical aspects might also be affected. This is of the highest interest since this phenomenon might also help to understand why, for instance, some serovars that are frequently isolated in chickens, such as *S*. Mbandaka or *S*. Livingstone [8] have such a low incidence in humans, despite not being poultry specific.

Unfortunately, not many works have been carried out in order to try to validate, at the species or subspecies level, if the development of increased stress resistance has a fitness and virulence cost for *Salmonella* [296,297]; further work is still required in order to corroborate it. In this sense, the results of Shah (2013), in spite of being obtained only with *S*. Enteritidis strains, are particularly interesting [298]. Thus, result obtained by this author indicate that whereas naturally virulent strains of this serovar (designated as high-pathogenicity or HP strains) were also those displaying a higher oxidative and osmotic stress resistance, low pathogenicity (LP) strains showed increased expression of the *tdc*, *gar*, and *gud* operons suggesting that the primary focus of these later strains could be survival and cell growth through enhanced nutrient acquisition rather than invasion and proliferation.

## 6. Concluding Remarks

From all that has been discussed in this review, one can conclude that, in most cases, the development of stress resistance responses imposes a fitness cost to *Salmonella* cells. By contrast, the impact of *Salmonella* stress resistance responses on the expression of virulence factors varies widely depending on the stressing agent and the virulence factor studied. In this sense, as discussed above, it should be noted that *Salmonella* possesses a plethora of virulence factors with a particularly tight and complex regulation. Furthermore, each one of these virulence factors plays a very well-defined role in *Salmonella* pathogenesis; since each one is only useful in a particular step during the infection process, virulence factors are induced or repressed sequentially during infection. Thus, for instance, increasing the expression of SPI-2 genes could be regarded as a phenomenon leading to an increase in *Salmonella* pathogenicity. Nevertheless, since the virulence factors encoded in SPI-2 will not help these bacteria to adhere to and invade the epithelial cells of the gut, they will probably not increase the chance of *Salmonella* cells to cause disease in a real scenario. What is more, they may even be counterproductive.

At this point it is important to remark that the impact of transient stress resistance responses to agents or technologies encountered in food and food processing environments on the expression of *Salmonella* virulence factors is normally not as relevant as the development of permanent responses since, once inside the host, *Salmonella* cells will have to sequentially cope with a number of different stresses that will trigger new adaptive responses and will re-define the virulence factors they express. In this sense, the transient stress responses that would probably exert a greater influence on *Salmonella* virulence are those affecting the bacterium’s resistance to the acid pH of the stomach, or those limiting the capability of *Salmonella* cells to adapt to other stresses that *Salmonella* might face within the host, such as bile salts or Fe starvation.

Having said this, studying the effect of transient stress responses on *Salmonella* virulence is still of the highest relevance for various reasons. First of all, it is important from a clinical point of view because *Salmonella* does indeed develop various transient stress resistance responses within the host that have been shown to be highly relevant for its pathogenesis. Secondly, these studies provide interesting and useful clues regarding the connections between different *Salmonella* regulation networks. Finally, they provide us with an idea of how cells that have developed permanent stress responses tend to behave.

From all the data presented above it can be concluded that stable variants/mutants with increased stress resistance display, in most cases, a decreased fitness cost and a lower virulence. It should be noted, however, that deletion mutants in certain genes involved in the development of stress resistance have been proven to be avirulent. This, in turn, reinforces the view that the risk of suffering salmonellosis would not only depend on the virulence of *Salmonella* cells (the amount of virulence factors expressed) but also on their ability to resist certain stresses once inside the host, as well as on the number of cells ingested (which, in turn, depends on the ability of *Salmonella* cells to grow and/or resist stresses in food). Therefore, the question whether the development of a stress resistance response would lead to an increase or decrease in the risk of suffering salmonellosis would not only be defined by the stress resistance response’s impact on the expression of virulence factors, but also on how it would impact the resistance to the stress and growth fitness. Depending on the level of induction of a stress response pathway, it is at least theoretically possible that the outcome might be completely different, ranging from no change to either an increase or a decrease in the probability (risk) of causing illness.

In addition, it should be noted that the different stress resistance (or ability to develop it) among *Salmonella* strains and/or serovars, and its impact on other aspects of *Salmonella* physiology, might help to explain why less than 20 serovars are responsible of more than 80% of all the cases reported, why some *S*. Enteritidis strains are more pathogenic that others or why some non-host-specific strains that are frequently isolated from animals and/or food products have a very low incidence of disease in humans.

In view of this, it is clear that more in-depth studies specifically tackling the impact of microbial stress resistance responses on other aspects of microbial physiology—especially, but not exclusively, growth fitness and virulence—are still required. Further studies on the pathogenicity of VBNC/persisters and biofilm cells should also be carried out. These studies will not only contribute to a more detailed knowledge of *Salmonella* physiology but may also improve our understanding of this genera’s ecology, while helping to improve current food preservation processes and risk assessment models.

## Figures and Tables

**Table 1:** Examples of stable *Salmonella* variants obtained after successive exposure to different selecting agents: impact on virulence, growth fitness and other phenotypical characteristics.

Selection Agent	Strain	Effect in Virulence	Effect in Growth Fitness	Other Characteristics	References
Acid Stress					
pH 2.5	*S*. Enteritidis 66045	Lower colonization of spleens and livers	Reduced growth rate and yields	-	[105]
pH 2.5	*S*. Typhimurium 30	Lower virulence	Reduced growth rate and yields	Increased heat resistance	[105]
**Osmotic stress**					
NaCl	*S*. Typhimurium NCCP10812	No changes in invasion	Not determined	Decreased atb resistance	[117]
NaCl	*S.* Enteritidis NCCP12243	Increased invasion	Not determined	Antibiotic susceptibility	[117]
**Oxidative stress, detergents and disinfectants**					
Blend of oxidizing compounds	*S.* Typhimurium SL1344	Decreased invasion	Reduced growth rate and yields	Decreased atb resistance Reduced motility	[136,137]
QA + FA + GA	*S.* Typhimurium SL1344	Decreased invasion	Reduced growth rate and yields	Decreased atb resistance Reduced motility	[136,137]
Phenolic tar acids-based disinfectant	*S.* Typhimurium SL1344	Decreased invasion	Reduced growth rate and yields	Decreased atb resistance Reduced motility	[136,137]
DTAC	*S.* Enteritidis ATCC 4931	Decreased invasion	Not determined	Fewer fimbriae	[185]
**Heat stress**					
55 °C	*S.* Typhimurium phage type l	Decreased virulence	Not determined	Increased roughness	[229]
**Non-Thermal Technologies**					
γ- radiation	*S*. Typhimurium phage type 2c	No change	Not determined	Increased roughness	[229]
γ- radiation	*S*. Typhimurium LT2	No change	Grows poorly in minimal media	Increased cell size	[236]
Ionizing radiation	*S.* Typhimurium ATCC 7823	Not determined	No change	-	[237,238]
Ionizing radiation	*S*. Newport ATCC 6962	Not determined	Reduced growth rate at 10–20 °C.	-	[237,238]
Ionizing radiation	*S.* Thompson ATCC 8391	Not determined	No change	-	[237,238]
Ionizing radiation	*S*. Heidelberg ATCC 8326	Not determined	No change	-	[237,238]
Ionizing radiation	*S.* Typhimurium strain RIA	Decreased virulence	Not determined	-	[239]
UV-C	*S.* Typhimurium (various strains)	Not determined	Not determined	Increased atb resistance	[284]
PEF	*S.* Typhimurium SL1344	Not determined	Not determined	-	[242]
PEF	*S.* Typhimurium CECT 443	Less virulent in *C. elegans*	Not determined	-	[243,244]
HHP	*S.* Typhimurium CECT 443	Less virulent in *C. elegans*	Not determined	-	[243,244]
